# The U.S. Food and Drug Administration Risk Assessment on Lead in Women’s and Children’s Vitamins Is Based on Outdated Assumptions

**DOI:** 10.1289/ehp.0900573

**Published:** 2009-03-25

**Authors:** Amir Miodovnik, Philip J. Landrigan

**Affiliations:** 1 Children’s Environmental Health Center; 2 Department of Community and Preventive Medicine and; 3 Department of Pediatrics, Mount Sinai School of Medicine, New York, New York, USA

**Keywords:** fetal and child neurobehavioral development, Food and Drug Administration, lead, provisional total tolerable intake levels, vitamins

## Abstract

**Background:**

Following a recent report of lead in certain commercial vitamin products, the U.S. Food and Drug Administration (FDA) conducted a nationwide survey to determine the Pb content in 324 multivitamin/mineral products labeled for use by women and children. The FDA compared estimated Pb exposures from each product with safe/tolerable exposure levels, termed provisional total tolerable intake (PTTI) levels, previously developed for at-risk population groups in 1992.

**Objective:**

We investigated the FDA’s conclusions that Pb concentrations in all vitamin products examined do not pose a hazard to health because they are below the PTTI levels for all groups considered.

**Discussion:**

For their initial estimations of PTTI levels, the FDA used a blood lead level (BLL) of 10 μg/dL as the threshold for adverse effects in children and in pregnant or lactating women. Studies have repeatedly linked chronic exposure to BLLs < 10 μg/dL with impairments in cognitive function and behavior in young children despite the absence of overt signs of toxicity. The FDA analysis also omitted any consideration of nonfood sources of Pb exposure, which is inconsistent with our current understanding of how most children develop elevated BLLs.

**Conclusion:**

We feel that based on these oversights, the FDA’s conclusions are unduly reassuring and that reconsideration of their current recommendations appears warranted.

In early 2007, following a report of elevated lead levels in certain commercial vitamin products, the U.S. Food and Drug Administration (FDA) conducted a nationwide survey in the United States to determine the Pb content in 324 multivitamin/mineral products (hereafter, vitamin products) labeled for use by women and children (U.S. FDA 2008). Four categories of vitamin products were examined, defined by the population groups for whom they are intended: young children (0–6 years of age), older children (≥ 7 years of age), pregnant or lactating women, and adult women.

To estimate the range of Pb exposures that might result from consumption of each vitamin product in the study, the FDA multiplied the amount of Pb in each product (the mass fraction) by the maximum recommended daily consumption, as indicated on the package label instructions (Mindak et al. 2008).

The amounts of Pb measured in these 324 vitamin products ranged from nondetectable to 2.88 μg/day in products intended for young children; from 0.0276 to 1.78 μg/day in vitamins for older children; from nondetectable to 8.97 μg/day in vitamins for pregnant and lactating women; and from 0.0146 to 4.92 μg/day in vitamins for other adult women.

To determine whether estimated Pb consumption from these vitamin products posed a hazard to human health, the FDA compared estimated Pb exposures with safe/tolerable exposure levels previously developed for age and sex groups, termed provisional total tolerable intake (PTTI) levels (Carrington and Bolger 1992). These PTTI levels were developed by the FDA in 1992 and are based on the assumption that food is the sole source of Pb exposure. Their goal was to predict the dietary intakes that would result in a blood lead level (BLL) of 10 μg/dL for children and pregnant or lactating women and of 30 μg/dL for other adults, including women of childbearing age. Accordingly, PTTI levels for dietary intake of Pb were set at 6 μg/day for young children and 25 μg/day for pregnant or lactating women.

Through this comparison, the FDA determined that five vitamin products would have resulted in exposures that exceeded 4 μg/day, the highest being 8.97 μg/day in a product labeled for pregnant or lactating women. The highest exposure estimate for children’s vitamins was 2.88 μg/day. Median Pb exposure was 0.576 μg/day. The FDA concluded that Pb concentrations in all vitamin products examined are below PTTI levels for all groups and that, therefore, none of the products pose a hazard to health.

## Misguided Assumptions

The FDA’s conclusion that Pb concentrations in commercial vitamin products pose no hazard to human health is based on outdated assumptions. It fails to consider recent literature on the potentially deleterious effects that low-level chronic Pb exposure may have on vulnerable populations.

### Improper use of the Centers for Disease Control and Prevention’s blood Pb action level of 10 μg/dL

In 1991, the U.S. Centers for Disease Control and Prevention (CDC) determined that primary prevention activities in children should begin at BLLs > 10 μg/dL (CDC 1991). It has become increasingly clear that the inverse relationship between blood Pb concentrations and health and developmental effects extends well below 10 μg/dL. Numerous epidemiologic studies over the past three decades have shown no evidence of a threshold for such effects, and, indeed, the indications are that the slope of the dose–response curve steepens as it approaches zero (Lanphear et al. 2005). Studies have repeatedly linked BLLs < 10 μg/dL in children 1–5 years of age with decreased IQ and impaired cognition (e.g., Bellinger and Needleman 2003), and associations with attention and behavior problems are becoming increasingly evident as well (Braun et al. 2006; Needleman et al. 1996). Strong and long-lasting neurobehavioral effects occur with BLLs as low as 2 μg/dL (Canfield et al. 2003; Jusko et al. 2008). A recent risk assessment by the California Environmental Protection Agency calculated that a 1-μg/dL change in BLL in the range of 1–10 μg/dL results in a population-level decrement of one IQ point (California Environmental Protection Agency 2009). Even a 1-point change in Full Scale IQ score, although within the standard error of an individual’s single measurement, is still highly significant on a population basis (Bellinger 2004).

### Failure to consider the heightened vulnerability of the developing fetus

The FDA analysis assumes that a blood level of 10 μg/dL is a threshold below which no adverse effects occur to the fetus and that a BLL of 30 μg/dL is a threshold level for adults, including non-pregnant women of childbearing age.

Maternal BLLs of approximately 10 μg/dL have been linked to increased risks of pregnancy hypertension, spontaneous abortion, and reduced neurobehavioral development in offspring. Somewhat higher maternal Pb levels have been linked to reduced fetal growth (Gardella 2001). Pb crosses the placenta from the maternal to the fetal circulation without impediment, and BLLs in mother and fetus are virtually identical. Developing fetuses and young children absorb Pb more readily than adults, and Pb enters the brain quite freely. Some of the neurobehavioral effects related to Pb exposure during fetal neurodevelopment appear to be permanent and persist into childhood (Bellinger et al. 1987; Gomaa et al. 2002; Shen et al. 1998). The FDA’s PTTI levels for Pb are at least one order of magnitude higher than the maximum allowable dose level of 0.5 μg/day, established by the California Environmental Protection Agency under the Safe Drinking Water and Toxic Enforcement Act for chemicals with known reproductive toxicity (California Environmental Protection Agency 2007).

### Using dietary Pb intake as the basis for daily Pb exposure

A second shortcoming in the FDA’s analysis is the assumption, embodied in the PTTI levels, that diet is the only source of exposure to Pb. In fact, there has been a marked reduction over the past 20 years of Pb exposure from food sources due to the elimination of solder in food and soft drink cans in the 1980s. Thus, today lead paint and dust account for up to 70% of elevated BLLs in U.S. children. The contribution of dust and soil is most critical for children 1–3 years of age, typically the age with the highest BLLs and greatest hand-to-mouth behaviors (Levin et al. 2008). The FDA analysis omits any consideration of these nonfood sources of exposure to Pb.

### Further shortcomings

A third shortcoming in the FDA analysis is its assumption that nonpregnant women of childbearing age may be considered equivalent to other adults in terms of their PTTI levels. This assumes that a woman ingests only a single source of Pb and that this exposure is terminated at the onset of pregnancy. The use of the adult PTTI level for pregestational women reflects the release of endogenous bone Pb that accumulated before pregnancy, but ignores the possible additive effects of fetal exposure to exogenous Pb from continued ingestion during pregnancy.

A fourth and final shortcoming in the FDA estimates is the assumption that all persons consume vitamins in doses that adhere to label instructions. In fact, previous studies of food consumption indicate a wide range of exposures in which some persons significantly exceed recommended consumption levels (National Research Council 1993). Higher exposures that result either from accidental overingestion or off-label use would lead to an increase in the overall Pb exposure risk.

## Conclusion

Reconsideration of the FDA’s conclusions and recommendations would appear warranted. The FDA should use the most current information available to guide future recommendations, especially when considering the health of children and pregnant women. Pb is a known neurotoxicant, and its presence in a readily available and widely consumed product such as vitamin supplements provides an unnecessary and preventable source of exposure.
